# Elevated Squamous Cell Carcinoma Antigen, Cytokeratin 19 Fragment, and Carcinoembryonic Antigen Levels in Diabetic Nephropathy

**DOI:** 10.1155/2017/5304391

**Published:** 2017-07-04

**Authors:** Jianzhong Chen, Feng Tao, Bin Zhang, Qingguang Chen, Yan Qiu, Qian Luo, Yanna Gen, Jiali Meng, Jue Zhang, Hao Lu

**Affiliations:** ^1^Department of Clinical Laboratory, Shuguang Hospital Affiliated to Shanghai University of Traditional Chinese Medicine, Shanghai, China; ^2^Department of Endocrinology and Metabolism, Shuguang Hospital Affiliated to Shanghai University of Traditional Chinese Medicine, Shanghai, China

## Abstract

**Objective:**

We aimed to explore whether squamous cell carcinoma antigen (SCC), cytokeratin 19 fragment (Cyfra21-1), neuron-specific enolase (NSE), and carcinoembryonic antigen (CEA) are elevated in diabetic nephropathy (DN) and the association between urinary albumin-to-creatinine ratio (UACR) and tumor markers in diabetic patients.

**Methods:**

Nondialysis patients with diabetes (*n* = 261) and 90 healthy controls were enrolled. DN was defined as an UACR ≥ 30 mg/g in the absence of a urinary tract infection or other renal abnormalities.

**Results:**

Patients with DN had significantly higher serum SCC, Cyfra21-1, and CEA levels than those with normoalbuminuria and healthy controls. The rates of positive SCC, Cyfra21-1, and CEA significantly increased with increasing urinary albumin excretion (all *P* for trend < 0.001). In contrast, NSE was not affected by DN. SCC, Cyfra21-1, and CEA were significantly and positively correlated with UACR. In logistic regression, after multivariable adjustment, increased UACR was associated with increased odds ratio of elevated tumor marker levels (all *P* for trend < 0.05).

**Conclusions:**

Serum levels of SCC, Cyfra21-1, and CEA are markedly increased with increasing urinary albumin excretion, which affects the specificity for diagnosis for lung cancer. Appropriate interpretation of tumor markers in diabetic patients is mandatory to avoid unnecessary and even hazardous biopsies.

## 1. Introduction

Diabetes is one of the most common chronic diseases in nearly all countries [1]. The prevalence of diabetes continues to increase worldwide and is estimated at 11.6% in China [2–4]. Accumulating evidence has shown that diabetes is associated with an increased risk for cancer [5, 6]. Therefore, the American Diabetes Association and the American Cancer Society recommended that all diabetic patients should undergo appropriate cancer screenings [7].

Moreover, diabetic patients have been reported to be at increased risk for lung cancer [8], which is the most common cancer and the leading cause of cancer-related deaths worldwide [9]. Squamous cell carcinoma antigen (SCC), cytokeratin 19 fragment (Cyfra21-1), neuron-specific enolase (NSE), and carcinoembryonic antigen (CEA) are widely used for screening, early detection, monitoring therapy efficacy, and defining prognosis of lung cancer [10, 11]; however, several studies have also shown that serum tumor marker levels are elevated in patients with chronic kidney disease (CKD) [12–15]. Such false positive elevation of tumor markers in patients with CKD might cause misuse of these tumor markers and even lead to unnecessary subsequent clinical procedures.

Diabetic nephropathy (DN), which is defined as increased urinary albumin excretion in the absence of other renal diseases [16], has emerged as a leading cause of CKD globally [17]. According to the American Diabetes Association, DN can be categorized into stages of microalbuminuria and macroalbuminuria based on the urinary albumin-to-creatinine ratio (UACR) [16, 18]. However, the effect of DN on serum tumor marker levels has not been studied. Moreover, in previous studies, CKD patients were divided into different groups according to their creatinine clearance rate (Ccr) [13, 14] or estimated glomerular filtration rate (eGFR) [15]. Of particular interest are the effects of different degrees of albumin excretion on these tumor markers in diabetic patients. Hence, we conducted this observational study to explore the association between UACR and these tumor marker levels in diabetic patients, in order to make better use of them in clinical practice.

## 2. Materials and Methods

### 2.1. Study Population

The study population consisted of 261 nondialysis type 2 diabetic patients with varying degrees of albumin excretion, who were inpatients of the Department of Endocrinology, Shuguang Hospital Affiliated to Shanghai University of Traditional Chinese Medicine between October 2015 and June 2016. Patients who met the diagnosis for type 2 diabetes based on the American Diabetes Association 2014 criteria, and did not accept dialysis, are eligible for inclusion. After systematic physical and radiological examinations, patients with any sign of malignancy, pregnancy, nondiabetic kidney disease, urinary tract infection, heart failure, hepatocirrhosis, or liver failure were excluded. Patients were divided into three groups according to UACR: normoalbuminuria group (DM, UACR < 30 mg/g, *n* = 96), microalbuminuria group (DN1, UACR ≥ 30 to< 300 mg/g, *n* = 88), and macroalbuminuria group (DN2, UACR ≥ 300 mg/g, *n* = 77). Following a careful clinical examination, 90 healthy age- and sex-matched volunteers were enrolled as the control group. The study protocol was approved by the Ethics Committee of Shuguang Hospital Affiliated to Shanghai University of Traditional Chinese Medicine. Written informed consent was obtained from all the participants.

### 2.2. Measurements

Detailed information regarding age, sex, medical history, and lifestyle-related risk factors were obtained by our clinical professionals. Current smoking was defined as having smoked at least 100 cigarettes in one's lifetime and currently smoking cigarettes [2]. Current drinking was defined as alcohol intake more than once per month during the past 12 months [2]. Weight and height were measured using a balance beam and a vertical ruler in light clothing and without shoes. Body mass index (BMI) was calculated as weight in kilograms divided by height in meters squared. Blood pressure (BP) was measured using a mercury sphygmomanometer, with participants in a seated position after 5 min of rest. Two BP readings were obtained 1 min apart, and the mean was calculated. Hypertension was defined by systolic blood pressure (SBP) ≥140 mmHg and/or diastolic blood pressure (DBP) ≥90 mmHg, or treatment with antihypertensive drugs.

Fasting venous blood and urine samples were collected from patients and controls in the morning and sent to clinical laboratories for measurement in time. SCC and CEA were measured using chemiluminescence immunoassays (Abbott i2000SR, Longford, Ireland); Cyfra21-1 and NSE were measured by electrochemiluminescence immunoassays (Modular e601; Roche Diagnostics, Mannheim, Germany). Fasting plasma glucose (FPG) was measured using hexokinase methods. HbA1C was measured using high-performance liquid chromatography with the VARIANTII Hemoglobin Testing System (Bio-Red laboratories). eGFR was calculated using the Modification of Diet in Renal Disease formula: eGFR (ml/min/1.73m^2^) = 186 × (serum creatinine × 0.011)^−1.154^ × (age) ^−0.203^ × (0.742 for women) [19]. The cutoff values for each tumor marker were 1.5 ng/ml for SCC, 5 ng/ml for CEA, 17 ng/ml for NSE, and 3.3 ng/ml for CYFRA21-1. Measured values greater than or equal to the cutoff value were defined as positive.

Urinary albumin and creatinine were measured using the nephelometry and sarcosine oxidase method (BECKMAN COULTER AU5800, Japan). Before examination was conducted, the patients were instructed to avoid exercise for 1 h. UACR was calculated by dividing urinary albumin by urinary creatinine and expressed in mg/g. DN was defined as an UACR of ≥30 mg/g in the absence of urinary tract infection or other renal abnormalities [16, 20].

### 2.3. Statistical Analysis

We performed the statistical analysis using IBM SPSS Statistics, Version 22 (IBM Corporation, Armonk, NY, USA). Two-sided *P* values <0.05 were considered significant. General characteristics were summarized as median with interquartile range (IQR) for continuous variables or as number with proportion for categorical variables. Kolmogorov-Smirnov tests and P-P plots were used to determine the normality of the data. To test for differences among the groups, Mann–Whitney *U* or Kruskal-Wallis tests were used for continuous variables with skewed distributions and Pearson chi-square tests for categorical variables. Spearman rank correlations with corresponding significance levels were evaluated to test the correlations between different variables.

Binary logistic regression analysis was conducted to determine the risk of elevated serum tumor marker levels for each category of UACR in diabetic patients, with normoalbuminuria (UACR < 30 mg/g) as the reference. Data were expressed as the odds ratio (OR) and 95% confidence intervals (CIs). Model 1 was unadjusted. Model 2 was adjusted for age, sex, BMI, duration of diabetes, FPG, HbA1C, eGFR, current smoking, current drinking, hypertension, and use of angiotensin-converting enzyme inhibitors (ACEI)/angiotensin receptor blockers (ARB).

## 3. Results

### 3.1. Clinical Characteristics of the Study Population

Baseline anthropometric and biochemical characteristics of the study population are summarized in Table 1. Among type 2 diabetic patients, 96 had normoalbuminuria, 88 had microalbuminuria, and 77 had macroalbuminuria. There were no significant differences in age and sex among the four groups. However, compared with the control group, diabetic patients with microalbuminuria and macroalbuminuria had significantly higher UACR, HbA1C, FPG, SBP, DBP, and BMI. These patients also had significantly lower eGFR. In addition, diabetic patients with macroalbuminuria had significantly higher UACR than those in the other groups, while FPG and BMI were not significantly different among the three diabetic groups.

### 3.2. Positive Rates and Serum Tumor Marker Levels

Serum tumor marker levels in the three diabetic groups and the healthy controls are shown in Table 2. Diabetic patients with microalbuminuria or macroalbuminuria had significantly higher serum SCC, Cyfra21-1, and CEA levels than those in the DM group and the control group. Moreover, diabetic patients with macroalbuminuria had significantly higher serum SCC and Cyfra21-1 levels than those in the other three groups. Meanwhile, the Cyfra21-1 levels in the three diabetic groups were significantly higher than those in the control group. By contrast, the four groups had comparable serum NSE levels.

**Figure 1:**
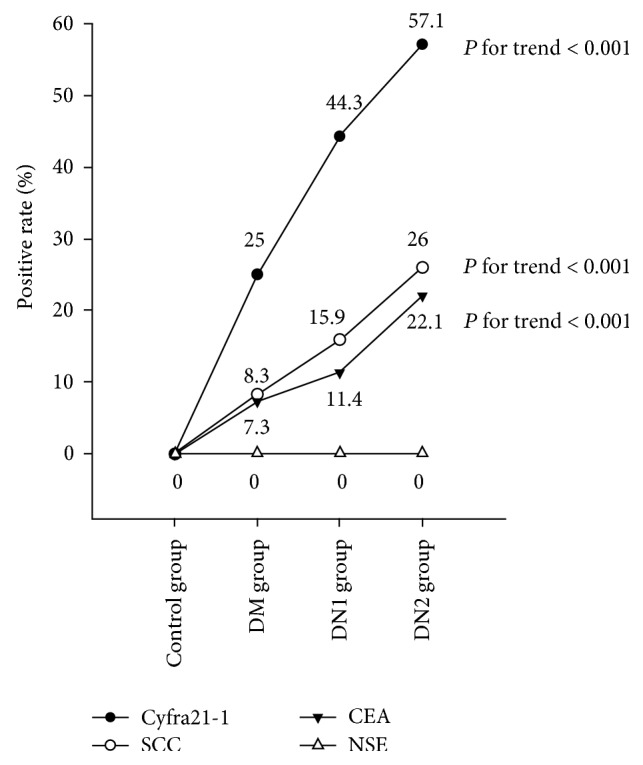
Positive rates of serum Cyfra21-1, SCC, CEA, and NSE levels in diabetic patients and healthy controls. DM: diabetes mellitus with normoalbuminuria; DN1: diabetes mellitus with microalbuminuria; DN2: diabetes mellitus with macroalbuminuria; SCC: squamous cell carninoma antigen; Cyfra21-1: cytokeratin 19 fragment antigen 21-1; NSE: neuron-specific enolase; CEA: carcinoembryonic antigen.

The positive rates for SCC, Cyfra21-1, and CEA significantly increased with increasing urinary albumin excretion (all *P* for trend < 0.001; Figure 1).

### 3.3. Correlations of Serum Tumor Marker Levels with the Urinary Albumin to Creatinine Ratio and Other Parameters in Diabetic Patients

Correlation results are presented in Table 3. SCC (*r* = 0.336), Cyfra21-1 (*r* = 0.299), and CEA (*r* = 0.348) were significantly and positively correlated with UACR. Significant negative correlations of eGFR with SCC, Cyfra21-1, and CEA were also observed. In addition, Cyfra21-1 was also significantly and positively correlated with age, duration of diabetes, and FPG. Significant positive correlations of CEA with duration of diabetes, FPG, and HbA1C were also observed. There were no associations of NSE with UACR and eGFR.

### 3.4. Association of UACR with Elevated Serum Tumor Marker Levels in Diabetic Patients

The binary logistic regression analyses (Table 4) showed that the risk of elevated SCC, Cyfra21-1, and CEA levels increased across the UACR categories (*P* for trend < 0.05 in every model). In an unadjusted model, compared with diabetic patients with normoalbuminuria, the ORs for positive SCC, Cyfra21-1, and CEA rates in patients with macroalbuminuria were 2.584 (95% CI 1.313, 5.083; *P* < 0.001), 4.000 (95% CI 2.097, 7.630; *P* < 0.001), and 2.783 (95% CI 1.356, 5.800; *P* < 0.01), respectively (Table 4, model 1). After adjusting for age, sex, BMI, duration of diabetes, FPG, HbA1C, eGFR, current smoking, current drinking, hypertension, and use of ACEI/ARB, ORs for elevated SCC, Cyfra21-1, and CEA levels were slightly attenuated but remained significant (Table 4, model 2).

## 4. Discussion

The present study showed that the serum levels of three tumor markers for lung cancer (SCC, Cyfra21-1, and CEA) were elevated in type 2 diabetic patients, especially those with DN. In addition, the positive rates for the three tumor markers gradually and markedly increased with increasing urinary albumin excretion. Binary logistic regression analyses indicated that UACR was an independent risk factor for elevated tumor markers in diabetic patients. In contrast, NSE was not affected by DN. To the best of our knowledge, this is the first study to explore the association between different degrees of albumin excretion and serum tumor marker levels in patients with type 2 diabetes.

Diabetes has been associated with an increased risk of lung cancer, which is now the most common cancer and the leading cause of cancer death worldwide [8, 9]. Serum SCC, Cyfra21-1, NSE, and CEA levels are now widely used to increase the diagnostic specificity for screening, early detection, monitoring therapy efficacy, and defining prognosis of lung cancer [11, 21]. However, accumulating evidence has shown that these tumor marker levels are elevated in patients with CKD [12–15]. Nomura et al. [13] reported significantly elevated serum SCC, Cyfra21-1, and CEA levels in patients with chronic renal failure, whereas NSE was not affected. Arik et al. [12] also reported increased serum SCC levels in nondialysis uremic patients.

However, few studies have explored the association between DN and tumor markers. Kashiwabara et al. [22] reported that serum Cyfra21-1 levels were higher in patients with DN than those in diabetic patients with normal renal function and healthy controls, which concurred with our results. Notably, this study was conducted 18 years ago and the definition of DN was based on abnormal urinary IgG-to-creatinine ratio (>1.1 mg/g). In the present analysis, we used UACR to define and categorize DN according to the American Diabetes Association criteria, which adds new and further evidence to the previous studies.

What is the possible underlying mechanism for the observed associations between serum tumor marker levels and UACR in diabetic patients? As point out previously, tumor marker levels may be modified by several tumor-independent physiological or pathological statuses that increase the synthesis (such as inflammation) and reduce its elimination (such as kidney or liver failure) [23]. First, albuminuria is a potent stimulus of mitochondrial dysfunction, inducing tubular injury and tubulointerstitial inflammation through oxidative stress [24]. In addition, urinary albumin excretion causes tubular lesions through activation of the HSP70-TLR4 axis in DN [25]. The tubular damage caused by albuminuria would lead to impaired excretion of these tumor markers. Second, many tumor markers are metabolized in the kidney [26]. Elevated levels of several tumor markers can be frequently detected in patients with impaired kidney function because their renal function is retarded [23]. Indeed, eGFR gradually decreased with increasing UACR in our study population, which could also be an important mechanism of the false positive elevations.

One interesting finding from the present study was that SCC, Cyfra21-1, and CEA levels were elevated even in the normoalbuminuric diabetic patients, with positive rates of 8.3%, 25.0%, and 7.3%, respectively. Previous studies have demonstrated that glomerular and tubular lesions occur early in diabetic patients in the absence of microalbuminuria [27–29]. Moreover, Surendar et al. [30] also found that cystetin C, an early indicator of renal impairment, was elevated even in patients with impaired glucose tolerance. Owing to the limited knowledge in this field, further investigation is warranted.

Taking into account the increased incidence of diabetes and DN, our study is of clinical importance. The cut-off values for SCC, Cyfra21-1, and CEA obtained from a normal population are not applicable to diabetic patients, especially those with DN. UACR is an important factor to be considered for prevention of diagnostic errors derived from false positive results. Considering NSE is not affected by DN, it may serve as a useful tool in the screening and early detection of lung cancer in diabetic patients.

Our study had some strengths. First, this study is the first to investigate the associations between UACR and tumor marker levels in diabetic patients. Second, strict quality control of the data is guaranteed by the fact that all information including potential confounders are collected by clinical professionals. However, our study also has some limitations. First, owing to the cross-sectional design, causal relationships between UACR and tumor marker levels could not be determined. Second, our sample size was relatively small. Further prospective and longitudinal studies with larger sample size and long-term follow-up are warranted. Third, among the 10 newly diagnosed diabetic patients, we used a single random measurement of UACR to diagnose DN rather than three measurements. However, recent data suggest that single urinary albumin measurements are accurate in predicting nephropathy [31] and have been used previously by other investigators [32].

## 5. Conclusion

In conclusion, serum SCC, Cyfra21-1, and CEA levels gradually and markedly increase with increasing urinary albumin excretion in diabetic patients. This affects the specificity for screening, early detection, monitoring therapy efficacy, and defining prognosis of lung cancer unless higher cutoff values are used. Appropriate interpretation of tumor markers in diabetic patients is mandatory to avoid unnecessary and even hazardous biopsies.

## Figures and Tables

**Table 1:** General characteristics of subjects included in the analysis.

Parameters	Control group	DM group	DN1 group	DN2 group
*N* (male/female)	90 (45/45)	96 (47/49)	88 (44/44)	77 (39/38)
UACR, mg/g	6.16 (4.91–7.96)	10.22 (6.76–14.07)^∗^	69.09 (42.10–136.47)^∗^^†^	796.80 (477.78–1874.10)^∗^^†$^
Age, yrs	63 (56–69)	63 (57–69)	64 (56–70)	63 (56–70)
Duration of diabetes, yrs	—	10 (6–16)	12 (8–16)	13 (9–18)
BMI, kg/m^2^	23.51 (22.09–25.51)	24.00 (21.84–27.17)^∗^	24.66 (22.98–27.60)^∗^	24.80 (23.15–27.80)^∗^
eGFR, ml/min/1.73m^2^	116.62 (100.71–136.27)	107.17 (92.23–125.11)^∗^	102.28 (85.56–120.36)^∗^	68.82 (45.12–87.16)^∗^^†$^
FPG, mmol/l	5.30 (5.00–5.50)	7.50 (6.30–8.60)^∗^	7.60 (6.40–9.10)^∗^	7.90 (6.50–9.20)^∗^
HbA1C, %	5.30 (5.10–5.50)	7.40 (6.50–8.20)^∗^	8.00 (7.10–9.20)^∗^^†^	8.10 (7.10–9.30)^∗^^†^
SBP, mmHg	120.0 (111.0–130.0)	130.0 (120.0–132.0)^∗^	130.0 (120.0–140.0)^∗^^†^	136.0 (130.0–146.0)^∗^^†$^
DBP, mmHg	75.0 (69.0–80.0)	80.0 (73.0–80.0)^∗^	80.0 (75.0–80.0)^∗^	80.0 (75.5–84.0)^∗^
Hypertension, %	—	21.9	31.8	42.9^†^
Use of ACEI/ARB, %	—	24.0	37.5^†^	44.2^†^
Current smoker, %	16.7	17.7	26.1	36.4^∗^^†^
Current drinker, %	8.9	10.4	14.8	26.0^∗^^†^

Data are summarized as median (interquartile range) for continuous variables or as number with proportion for categorical variables. DM: diabetes mellitus with normoalbuminuria; DN1: diabetes mellitus with microalbuminuria; DN2: diabetes mellitus with macroalbuminuria; UACR: urinary albumin to creatinine ratio; BMI: body mass index; eGFR: estimated glomerular filtration rate; FPG: fasting plasma glucose; HbA1C: glycated hemoglobin; SBP: systolic blood pressure; DBP: diatolic blood pressure; ACEI: angiotensin-converting enzyme inhibitors; ARB: angiotensin receptor blockers. ^∗^*P* < 0.05 versus control group; ^†^*P* < 0.05 versus DM group; ^$^*P* < 0.05 versus DN1 group.

**Table 2:** Serum tumor marker levels in diabetic patients and controls.

Tumor markers	Control group	DM group	DN1 group	DN2 group
*N*	90	96	88	77
SCC	0.50 (0.10–0.90)	0.60 (0.20–0.90)	0.80 (0.40–1.10)^∗^^†^	1.25 (0.58–1.80)^∗^^†$^
Cyfra21-1	1.70 (1.28–2.15)	2.20 (1.80–3.28)^∗^	2.95 (2.10–3.90)^∗^^†^	3.50 (2.60–4.50)^∗^^†$^
NSE	6.32 (5.72–7.35)	6.42 (5.46–7.26)	6.53 (5.04–8.65)	6.77 (5.67–8.75)
CEA	1.67 (1.12–2.57)	2.02 (1.46–3.11)	2.71 (1.86–4.02)^∗^^†^	3.43 (2.43–4.89)^∗^^†^

Data are summarized as median (interquartile range) for continuous variables. DM: diabetes mellitus with normoalbuminuria; DN1: diabetes mellitus with microalbuminuria; DN2: diabetes mellitus with macroalbuminuria; SCC: squamous cell carninoma antigen; Cyfra21-1: cytokeratin 19 fragment antigen 21-1; NSE: neuron specific enolase; CEA: carcinoembryonic antigen. ^∗^*P* < 0.05 versus control group; ^†^*P* < 0.05 versus DM group; ^$^*P* < 0.05 versus DN1 group.

**Table 3:** Spearman correlation coefficients between serum tumor markers and measured parameters.

	SCC	CA211	CEA
Age, yrs	0.061	0.132^∗^	0.023
BMI, kg/m^2^	0.046	0.076	0.050
Duration of diabetes	0.051	0.255^∗∗∗^	0.150^∗^
FPG, mmol/l	0.061	0.146^∗^	0.247^∗∗^
HbA1C, %	0.076	0.134	0.234^∗∗^
UACR	0.336^∗∗∗^	0.299^∗∗∗^	0.348^∗∗^
eGFR, ml/min/1.73m^2^	−0.195^∗∗^	−0.249^∗∗∗^	−0.142^∗^

Data were spearman correlation coefficients. ^∗^*P* < 0.05; ^∗∗^*P* < 0.01; ^∗∗∗^*P* < 0.001. BMI: body mass index; FPG: fasting plasma glucose; HbA1C: glycated hemoglobin; UACR: urinary albumin to creatinine ratio; eGFR: estimated glomerular filtration rate. SCC: squamous cell carninoma antigen; Cyfra21-1: cytokeratin 19 fragment antigen 21–1; CEA: carcinoembryonic antigen.

**Table 4:** Associations of urinary albumin-to-creatinine ratio with tumor marker status (positive or negative) in diabetic patients.

	Urinary albumin-to-creatinine ratio			
	<30	30–299	≥300	*P* for trend
SCC positive				
Model 1	Ref.	1.023 (0.506–2.067)	2.584 (1.313–5.083)^∗∗∗^	0.002
Model 2	Ref.	1.113 (0.815–3.569)	2.552 (1.297–5.021)^∗∗^	0.003
Cyfra21-1 positive				
Model 1	Ref.	2.388 (1.278–4.460)^∗∗^	4.000 (2.097–7.630)^∗∗∗^	<0.001
Model 2	Ref.	1.952 (1.010–3.774)^∗^	2.443 (1.054–5.664)^∗^	0.026
CEA positive				
Model 1	Ref.	0.996 (0.362–1.748)	2.783 (1.336–5.800)^∗∗^	0.006
Model 2	Ref.	1.275 (0.440–3.691)	2.299 (1.077–4.907)^∗^	0.035

Data were odds ratio (95% confidence interval). ^∗^*P* < 0.05; ^∗∗^*P* < 0.01; ^∗∗∗^*P* < 0.001. SCC: squamous cell carninoma antigen; Cyfra21-1: cytokeratin 19 fragment antigen 21-1; CEA: carcinoembryonic antigen. Model 1 was unadjusted. Model 2 included terms for age, sex, BMI, duration of diabetes, FPG, HbA1C, eGFR current smoking and current drinking, hypertension, and use of ACEI/ARB. SCC positive was defined as serum SCC level ≥ 1.5 ng/ml; Cyfra21-1 positive was defined as serum Cyfra21-1 level ≥ 3.3 ng/ml; CEA positive was defined as serum CEA level ≥ 5.0 ng/ml.
